# Editorial: Germplasm conservation and semen banking in livestock: strategies, challenges, and applications

**DOI:** 10.3389/fvets.2026.1878394

**Published:** 2026-06-09

**Authors:** Sourabh Deori

**Affiliations:** Indian Council of Agricultural Research Research Complex for North Eastern Hill Region (ICAR RC NEH), Umiam, India

**Keywords:** challenges, conservation, germplasm, livestock, semen banking

The conservation of livestock genetic resources has become one of the most critical priorities in modern animal agriculture. The accelerated genetic dilution of indigenous breeds, the emergence of transboundary diseases, climate instability, and the increasing dominance of high-yielding commercial germplasm threaten the long-term sustainability of global livestock biodiversity. Indigenous and locally adapted breeds possess unique genetic traits related to disease resistance, climatic adaptability, fertility, feed efficiency, and survival in low-input production systems. Preservation of these valuable genetic resources is therefore essential not only for maintaining biodiversity but also for ensuring future food security, sustainable livestock production, and resilience against environmental and epidemiological challenges.

In this context, germplasm conservation and semen banking have emerged as indispensable tools for safeguarding animal genetic resources. Advances in reproductive biotechnology, cryobiology, molecular genetics, and assisted reproductive technologies have substantially improved the ability to preserve and utilize valuable livestock germplasm. However, challenges related to cryoinjury, oxidative stress, poor post-thaw fertility, species-specific variability in cryotolerance, and limited understanding of molecular fertility mechanisms continue to restrict the efficiency of long-term germplasm preservation systems.

The Research Topic “*Germplasm conservation and semen banking in livestock: strategies, challenges, and applications*” was therefore conceptualized to provide an interdisciplinary scientific platform that addresses these challenges through innovative research in reproductive biotechnology, gamete preservation, embryo cryobiology, molecular fertility regulation, and advanced cryoprotective strategies. This Research Topic specifically aimed to integrate fundamental reproductive biology with practical applications in livestock breeding, artificial insemination, embryo transfer, and conservation programs.

## Aims and objectives of this Research Topic

The major objective of this Research Topic was to generate scientific knowledge to improve the efficiency, reliability, and practical applicability of livestock germplasm conservation systems. This Research Topic focused particularly on:

1. Developing innovative cryopreservation approaches to improve the post-thaw viability and fertility of spermatozoa, oocytes, and embryos.
2. Understanding the molecular, cellular, endocrine, and metabolic mechanisms that govern gamete cryotolerance and reproductive performance.
3. Identifying safer and more efficient cryoprotectants that can minimize cryoinjury and oxidative stress.
4. Optimizing *in vitro* fertilization and embryo production systems using preserved gametes and embryos.
5. Strengthening livestock conservation programs through advanced reproductive technologies and molecular reproductive tools.
6. Supporting livestock breeding and genetic improvement programs through improved fertility preservation strategies and biomarker discovery.

The four manuscripts published under this Research Topic collectively address these objectives and significantly contribute to advancing livestock reproductive biotechnology and conservation science.

## Molecular regulation of fertility and reproductive efficiency

One of the central objectives of the Research Topic was to understand the molecular and physiological mechanisms associated with reproductive performance and gamete quality. The article entitled “*Integrative analysis of the hypothalamic-pituitary-testicular axis reveals molecular mechanisms underlying sperm motility differences in Landes ganders*” (Liu et al.) directly addressed this objective by investigating the endocrine and transcriptomic regulation of sperm motility in ganders.

The study employed an integrative analysis of the hypothalamic–pituitary–testicular (HPT) axis and identified key genes, signaling pathways, and hormonal interactions associated with differences in sperm motility and male fertility. By linking molecular pathways with reproductive phenotypes, the study contributes substantially to our understanding of fertility regulation mechanisms in avian species.

Importantly, the knowledge generated from this work has practical implications for livestock breeding and germplasm conservation programs. Identification of fertility-associated genes and molecular biomarkers can support the following:

- Marker-assisted selection of breeding males with superior reproductive performance,
- Early fertility prediction in breeding programs,
- Development of genomic selection tools,
- Improvement of protocols for evaluating semen quality in poultry species.

These applications are particularly valuable for conserving elite and indigenous avian germplasm, where maintaining reproductive efficiency is essential for sustainable breeding and semen banking programs.

## Advancement of novel cryoprotective strategies

Cryoinjury and oxidative damage are major obstacles that limit the successful preservation of gametes and embryos. Addressing these issues was one of the primary objectives of this Research Topic. The article “*Research progress on novel bionic cryoprotectants in gamete and embryo cryopreservation*” (Yang et al.) made a highly significant contribution to this goal.

The review comprehensively summarized recent advances in biomimetic and bioinspired cryoprotectants, including:

- Antifreeze proteins,
- Nanoparticle-based cryoprotectants,
- Synthetic polymers,
- Naturally derived biomolecules,
- Cell membrane stabilizers.

These bionic cryoprotectants mimic natural biological defense systems against cold stress and ice crystal formation, thereby reducing cellular injury during freezing and thawing.

The article provides a strong scientific foundation for developing safer and more efficient cryopreservation systems with lower cytotoxicity than conventional cryoprotective agents such as glycerol and DMSO. The practical application of this knowledge includes:

- Formulation of next-generation semen extenders,
- Improvement of embryo vitrification protocols,
- Reduction of oxidative and osmotic stress during freezing,
- Enhancement of post-thaw fertility and embryo survival,
- Development of species-specific cryopreservation protocols for endangered livestock breeds.

The review also highlights the future potential of integrating nanotechnology and biomimetic materials into livestock cryobiology, which could revolutionize germplasm banking systems worldwide.

## Optimization of *in vitro* embryo production systems

Another important objective of the Research Topic was to improve reproductive competence and developmental efficiency of cryopreserved gametes and embryos under assisted reproductive systems. The manuscript “*Comparative study on in vitro fertilization, cleavage and blastocyst development of post-warmed vitrified cattle oocytes in different media*” (Sebopela et al.) addressed this objective through evaluation of the post-thaw developmental competence of vitrified bovine oocytes.

The study demonstrated that culture conditions and fertilization media significantly influence the following:

- Fertilization rates,
- Cleavage efficiency,
- Blastocyst development,
- Embryo quality following vitrification and warming.

These findings are highly relevant to cattle embryo production and embryo banking programs. Optimization of culture systems can improve the success rates of *in vitro* fertilization and embryo transfer technologies, particularly when using cryopreserved oocytes.

The practical applications of this research include:

- Standardization of IVF media for cryopreserved bovine oocytes,
- Improvement of embryo production efficiency in cattle breeding programs,
- Establishment of large-scale embryo banking systems,
- Conservation of elite female germplasm,
- Increased genetic dissemination through advanced reproductive technologies.

This study therefore contributes directly to improving the reliability of embryo preservation systems for livestock conservation and genetic improvement programs.

## Mitochondrial protection and oxidative stress regulation during cryopreservation

Oxidative stress and mitochondrial dysfunction are among the most important causes of sperm cryodamage. Addressing oxidative injury through novel additives and metabolic regulation strategies was another primary objective of this Research Topic. The article “*Protective roles of melatonin regulated mitochondrial DNA methylation and respiratory metabolism in ram sperm cryopreservation*” (Hu et al.) contributed significantly in this area.

The study demonstrated that melatonin exerts cytoprotective effects during ram sperm cryopreservation through regulation of the following:

- Mitochondrial DNA methylation,
- Respiratory metabolism,
- Oxidative stress pathways,
- Cellular energy homeostasis.

The findings showed improved sperm functionality and cryosurvival following melatonin supplementation, highlighting its potential as an effective antioxidant additive in semen extenders.

The practical implications of this work are extensive and include:

- Development of antioxidant-enriched semen extenders,
- Improvement of post-thaw sperm motility and viability,
- Enhancement of fertility following artificial insemination,
- Reduction of mitochondrial damage during freezing,
- Improvement of semen preservation protocols for small ruminants and other livestock species.

Furthermore, this study advances our understanding of epigenetic and mitochondrial regulation during cryopreservation, opening new possibilities for precision cryobiology and fertility preservation research.

## Collective contribution to germplasm conservation

Taken together, the manuscripts published under this Research Topic successfully fulfill the major objectives envisioned during its conception. The studies integrate molecular reproductive biology, cryobiotechnology, antioxidant research, embryo culture optimization, and biomimetic preservation strategies to address critical limitations in livestock germplasm conservation systems.

Importantly, the knowledge generated through these studies can be translated into practical applications such as:

- Improved semen and embryo banking protocols,
- Enhanced artificial insemination efficiency,
- Fertility biomarker identification,
- Advanced reproductive management systems,
- Conservation of endangered and indigenous breeds,
- Sustainable livestock breeding and genetic improvement programs.

The studies collected here also demonstrate how multidisciplinary integration involving omics technologies, reproductive physiology, molecular biology, nanotechnology, and cryobiology can accelerate progress in animal genetic resource conservation.

Despite considerable advancements, important challenges remain, including species-specific variability in cryotolerance, limited field-level application of advanced technologies, high preservation costs, and an incomplete understanding of fertility-associated molecular pathways. Future research integrating artificial intelligence-based fertility prediction, precision cryobiology, epigenetics, metabolomics, and nanobiotechnology may further enhance the efficiency of systems for preserving livestock germplasm.

In conclusion, this Research Topic provides a comprehensive scientific resource for reproductive biotechnologists, veterinarians, livestock breeders, and conservation scientists working toward the sustainable management of animal genetic resources. The collective findings not only advance the scientific understanding of reproductive preservation mechanisms but also provide practical strategies for improving semen banking, embryo preservation, fertility management, and the long-term conservation of valuable livestock germplasm for future generations.

